# A Cross-Sectional Study to Assess the Awareness and Health-Seeking Behavior Regarding Animal Bites Among Residents of Urban Slums in the Field Practice Area of a Medical College in Eastern India

**DOI:** 10.7759/cureus.96525

**Published:** 2025-11-10

**Authors:** Nirmalya Mohapatra, Krishna Mishra, Ipsita Debata, Angana Ray, Spandan Mishra

**Affiliations:** 1 Community Medicine, Kalinga Institute of Medical Sciences, Bhubaneswar, IND

**Keywords:** animal bite exposure, awareness, health-seeking behavior, rabies, urban slums

## Abstract

Introduction: Rabies is one of the oldest viral diseases caused by the rabies virus, belonging to the Mononegavirales order, Rhabdoviridae family, and *Lyssavirus *genus. Rabies is a zoonotic disease affecting both public health and the livestock industry in India. Rabies causes incurable viral encephalitis, and its progressiveness is considered to be extremely fatal. General awareness regarding the disease, the sequel, prevention, and treatment plays an important role in curbing deaths due to rabies. Now, with an increase in the number of street dogs, it has become increasingly important for residents to be aware of the disease and the need to seek timely medical care in case of animal bite exposure. With this background, the current study was planned with the objectives to assess knowledge and awareness regarding rabies and health-seeking practices among the urban adults residing in urban slums (study area).

Methods: This was a community-based, cross-sectional study conducted in urban slums under the field practice area of a tertiary medical college and teaching hospital in Odisha. Using stratified random sampling with proportional allocation, 140 study participants were included who gave written informed consent. A semi-structured, pre-tested questionnaire was used for data collection, and the knowledge and attitude were scored. Data were coded, analyzed using Epi Info v3.5.1 (Centers for Disease Control and Prevention, Atlanta, Georgia, USA), and represented in frequencies (percentages). The chi-square and Fisher's exact tests, wherever applicable, were used as tests of significance.

Results: Around 72.1% of the study participants had adequate knowledge, and 63.0% had good health-seeking practices following an animal bite. It was found that educational status (p < 0.001) and socioeconomic status (p = 0.003) were statistically significantly associated with knowledge about rabies amongst the study participants. Other variables, like first-aid sought after a bite, place of seeking treatment, observation of the biting animal, time of seeking anti-rabies vaccine (ARV) following an animal bite, and recognizing the category of wound correctly, were found to be significantly associated with various practices following an animal bite (p < 0.001).

Conclusion: The level of awareness was found to be lower among the adults residing in urban slums. The practices were found to be good among a few, and the health-seeking behavior was also found to be low within 24 hours following animal bite exposure. There were various sociodemographic variables affecting the level of awareness. Several pet-related factors, like owner of a pet, first-aid after a bite, and the place of getting treated, were found to be associated with the practices among the study participants.

## Introduction

Rabies is a zoonosis affecting the human population after being bitten by a rabid animal. The fatality rate depends on the site, severity, age of the victim, category of the exposure, and the time and place of treatment seeking. Usually affecting the poorest communities, rabies is a neglected zoonotic tropical disease. The disease marks its existence on the entire globe (around 150 countries), except for a few areas like the Democratic People's Republic of Korea, the Maldives, and Timor-Leste [1]. India is one of the nations with the largest number of stray dogs worldwide. Indians are at a high risk of contracting rabies because these stray dogs are not vaccinated [2]. As per the recent data available, only around 57% of the stray dogs in Odisha are vaccinated. Under the National Action Plan for Dog-Mediated Rabies Elimination (NAPRE), India's National Rabies Control Program (NRCP) aims to eradicate dog-mediated rabies by 2030. The incidence of rabies in India has decreased from 2.36 to 0.41 cases per 10 million people, but between 2005 and 2020, more than 75% of cases were reported mainly in five states (West Bengal, Andhra Pradesh, Maharashtra, Karnataka, and Delhi), with West Bengal accounting for 43% of all cases. In 2021, human rabies became a disease that needed to be reported in order to improve data and inform strategy [3,4].

Intending to achieve zero rabies-related deaths by 2030, the Odisha government made human rabies a notifiable disease in September 2023. This enhances surveillance and control methods by requiring all healthcare establishments to report rabies cases promptly. To improve diagnostic capabilities, the state is establishing a rabies diagnostic laboratory and emphasizing the importance of prompt post-exposure prophylaxis. Following Karnataka, Andhra Pradesh, Kerala, Tamil Nadu, Madhya Pradesh, Punjab, Gujarat, and Maharashtra, Odisha is now the 12th state to declare rabies a notifiable disease [5]. A multi-centric Indian study in 2005 reported that the majority of rabies deaths in India occurred in children aged five to 15 years and those from lower socioeconomic backgrounds [6]. The prevention and management of rabies in both humans and animals depend heavily on community awareness of the disease and the health-seeking behavior of individuals. Many Indian studies conducted in various states have discovered that people's knowledge of the rabies virus and how to treat animal bite wounds after a dog bite varies [7-10].

Slums are areas characterized by open dumping of waste and improper waste segregation practices, which attract a large number of stray dogs to the area. This creates a potentially dangerous situation for the large number of unsupervised children who become more prone to dog attacks as compared to adults. In this setting, knowledge and awareness regarding the disease "rabies" and utilization of the available vaccine and immunoglobulin, which are free of cost, for the prevention of rabies, are one of the biggest steps towards avoiding mortality. Designing successful prevention strategies requires a thorough understanding of the community's knowledge, attitudes, and practices surrounding rabies management [11].

Deeply ingrained cultural and religious beliefs related to rabies, a dysfunctional health and civil registration system, a misplaced trust in home remedies and traditional medicine systems, and other factors all make it difficult to implement timely and appropriate medical interventions to prevent rabies in India [12-15]. There is a paucity of literature on the mentioned topic in the study area of Odisha. Hence, this study was undertaken in the urban slums to assess knowledge and awareness regarding rabies and health-seeking practices among the selected adult residents of the urban slums.

A part of the study was previously presented as an oral paper at the 23rd National Conference of the Association for Prevention and Control of Rabies in India (APCRICON 2023) on July 9, 2023, at Lucknow, Uttar Pradesh.

## Materials and methods

Study type, design, and study area

This community-based, cross-sectional study was conducted among the population residing in urban slums within the field practice area of the Urban Health and Training Center (UHTC) of the Department of Community Medicine at Kalinga Institute of Medical Sciences in Bhubaneswar, Odisha.

Study duration

The study was conducted between April 2024 and June 2024.

Study setting

The study was conducted in five slums under the UHTC of the Department of Community Medicine at Kalinga Institute of Medical Sciences in Bhubaneswar, Odisha. The slums comprised a total of 3,120 households.

Study population

The study included consenting adults aged 18 years and above, of both genders, who had been residing in urban slums for more than six months. The houses that were locked after two visits and those with adults having cognitive impairment were excluded.

Sample size calculation

The sample size was estimated to be 136 based on a previous study by Sivagurunathan et al. [16], who reported a prevalence of rabies awareness as 76% among adults residing in urban slums at a 95% confidence interval, 10% relative precision, and a 10% non-response rate. The final sample size was rounded up to 140.

Sampling technique

The study area comprised five slums within the field practice area of UHTC, which catered to 3,120 households. Using stratified random sampling with proportional allocation according to the number of households in each slum, 140 households were selected for the study. The list of all households in the study area, maintained by the UHTC, was referred to for this. Systematic random sampling was used within each stratum: the first household was selected by lottery, and each kth household (22nd household) was added until the goal was reached. The sampling interval was determined by dividing the total number of households by the allotted sample size. One adult per household was chosen at random till the desired sample size was met. Non-responding households were revisited twice; persistent non-responders were replaced by the next household on the list.

Data collection tool

The data were collected using a pre-tested, semi-structured questionnaire (see Appendix A). Standardization and necessary modifications of the questionnaire were made through pilot testing with 20 participants; the results of this testing were not included in the final analysis. The questionnaire consisted of three parts. Part A contained the sociodemographic details of the study participants. The socioeconomic status was classified as per the modified BG Prasad scale [17]. Part B included questions assessing knowledge regarding rabies (epidemiology, symptoms, and transmission), first aid measures, and vaccination after an animal bite. A total of 27 items covering rabies etiology, transmission, clinical features, prognosis, and prevention were used to gauge knowledge and awareness. The maximum possible score was 27, with each right answer receiving a score of one and incorrect or "don't know" answers receiving a score of zero. A score of 14 or higher indicated adequate knowledge, while a score of less than 14 indicated inadequate knowledge. 

Part C consisted of six essential items on bite management, treatment seeking, and wound management, which were used to evaluate practices. The maximum practice score was six, with each appropriate practice receiving one point. Respondents with a score of at least three were deemed to have good practices, whereas those with a score below three were considered to have poor practices.

The data were collected by interviewing one adult per household in the local language, after obtaining valid consent.

Data analysis

The data were analyzed using Epi Info v3.5.1 (Centers for Disease Control and Prevention, Atlanta, Georgia, USA). Descriptive data were presented in terms of frequencies, percentages, and means. The chi-square/Fisher’s exact tests were used as applicable to test the association between sociodemographic factors and knowledge and practices with respect to rabies. A statistical significance was established at a p-value less than 0.05.

Ethical consideration

The study commenced after obtaining institutional ethical clearance (approval no.: KIIT/KIMS/IEC/1879/2024). Written informed consent was obtained from all participants, and confidentiality was maintained throughout the process.

## Results

Sociodemographic profile

A total of 140 participants were included in the study. Around 70% (98) were male, and 52.1% (73) were above 40 years of age. The mean age was 40.4 years. Around 40.7% (57) were educated till middle school, and 44.2% (62) owned a pet (either dogs, cats, or cows).

Knowledge about rabies

Around 72.1% (101) had adequate knowledge and awareness of rabies. The educational status (χ² = 27.115, p < 0.001) and socioeconomic status (χ² = 16.379, p = 0.003) of the participants were significantly associated with knowledge and awareness (Table 1).

**Table 1:** Association between sociodemographic factors and knowledge and awareness outcomes among study participants (n=140) Chi-square test/Fisher's exact test was used for testing association. OBC: Other backward class; SC: Scheduled caste; ST: Scheduled tribe

Variable	Category	Adequate Knowledge (n=101), N (%)	Inadequate Knowledge (n=39), N (%)	χ² Value	P-Value
Age group (years)	<40 (n=67)	49 (73.1)	18 (26.9)	0.063	0.802
	≥40 (n=73)	52 (71.2)	21 (28.8)		
Sex	Male (n=98)	71 (72.4)	27 (27.6)	0.0152	0.901
	Female (n=42)	30 (71.4)	12 (28.6)		
Caste	General (n=91)	67 (73.6)	24 (26.4)	4.846	0.183
	SC (n=13)	06 (46.2)	07 (53.8)		
	ST (n=09)	06 (66.7)	03 (33.3)		
	OBC (n=27)	22 (81.5)	5 (18.5)		
Pet ownership	Yes (n=62)	48 (77.4)	14 (22.6)	1.542	0.214
	No (n=78)	53 (67.9)	25 (32.1)		
Educational status	Illiterate (n=11)	0 (0.0)	11 (100.0)	27.115	<0.001
	Below primary (n=14)	08 (57.1)	06 (42.9)		
	Middle school (n=57)	46 (80.7)	11 (19.3)		
	High school (n=50)	40 (80.0)	10 (20.0)		
	Intermediate (n=8)	07 (87.5)	01 (12.5)		
Socioeconomic status	Upper (n=20)	18 (90.0)	02 (10.0)	16.379	0.003
	Upper Middle (n=30)	24 (80.0)	06 (20.0)		
	Lower Middle (n=35)	23 (65.7)	12 (34.3)		
	Upper Lower (n=30)	17 (56.7)	13 (43.3)		
	Lower (n=25)	19 (76.0)	06 (24.0)		

The health-seeking behavior was considered to be good if the victim sought medical help within 24 hours of animal bite exposure. Good health-seeking practices were observed among 63% (88) of the study participants, while 37% (52) had poor practices after an animal bite. These outcomes were found to be significantly associated (p < 0.001) with the majority of the variables, like first-aid after a bite, place of seeking treatment, action taken for the biting dog, awareness of anti-rabies vaccine (ARV) timing, action taken for a rabid pet, and recognition of the category of bite (Table 2).

**Table 2:** Association between various factors and health-seeking practices (good vs. poor) among study participants (n=140) Chi-square test was used for testing association. ARV: Anti-rabies vaccine

Variable	Response	Good Practices (n=88), N (%)	Bad Practices (n=52), N (%)	χ² Value	P-Value
First aid after a bite	Washed wound with soap/water	60 (68.2)	12 (23.1)	30.715	<0.001
	Used antiseptic (alcohol/iodine)	10 (11.4)	6 (11.5)		
	Applied salt/oil/herbal remedies	12 (13.6)	22 (42.3)		
	Did nothing	6 (6.8)	12 (23.1)		
Place of seeking treatment	Government hospital	55 (62.5)	10 (19.2)	35.699	<0.001
	Private hospital	20 (22.7)	10 (19.2)		
	Household remedies/traditional healer	8 (9.1)	18 (34.6)		
	Quack/no treatment	5 (5.7)	14 (26.9)		
Action for a biting dog	Observed for 10 days	65 (73.9)	15 (28.8)	33.126	<0.001
	Released if vaccinated	12 (13.6)	8 (15.4)		
	Killed immediately	8 (9.1)	20 (38.5)		
	Other/don’t know	3 (3.4)	9 (17.3)		
Awareness of ARV timing	After bite/exposure	72 (81.8)	22 (42.3)	23.206	<0.001
	Before exposure only	10 (11.4)	20 (38.5)		
	Don’t know	6 (6.8)	10 (19.2)		
Action if the pet is rabid	Consult veterinarian	68 (77.3)	18 (34.6)	25.259	<0.001
	Kill the animal	12 (13.6)	22 (42.3)		
	Separate/isolate	5 (5.7)	8 (15.4)		
	Don’t know/other	3 (3.4)	4 (7.7)		
Exposure recognition	Recognized category II/III as risky	74 (84.1)	20 (38.5)	30.847	<0.001
	Recognized category I only/not sure	14 (15.9)	32 (61.5)		

**Figure 1:**
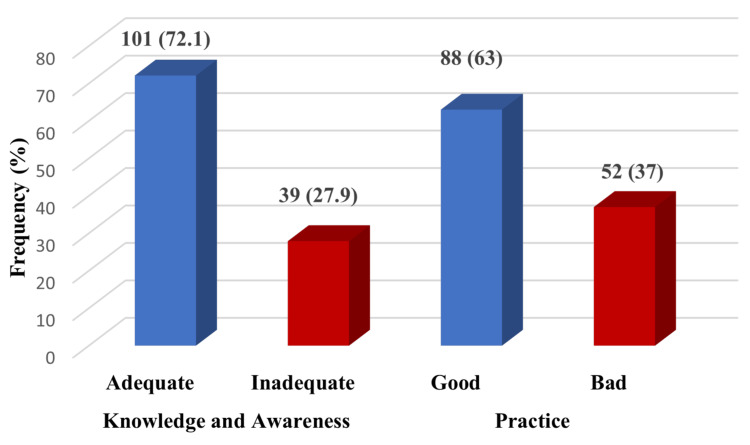
Classification of knowledge and practice outcomes among study participants (n=140)

The overall classification of knowledge and practice among the study participants is depicted in Figure 1.

## Discussion

The present study, which was conducted among 140 adult residents of urban slums, depicted that around 72.1% of the study participants had adequate knowledge about rabies, which is lower than the results of the study conducted by Dutta et al. in the rural community of New Delhi that showed around 93.4% had knowledge about rabies [18]. In the current study, around 68.2% of the participants knew that in case of exposure to an animal bite, the wound should be washed with soap and water, and did the same. This finding is better than the findings of the study conducted by Sivagurunathan et al. in Tamil Nadu, where only around 37.6% knew that the wound should be washed with soap and water [16]. Another similar study conducted by Verma et al. in Uttar Pradesh depicted that washing and first aid were done by only 34.6% of the study participants [11].

A study conducted by Tiwari et al. in Western India reported that family size and ownership of a pet (cat or dog) were associated with knowledge about rabies, whereas in the present study, educational status, socioeconomic status, and owning a pet were found to have a significant association with knowledge and practices about rabies [19]. In a study conducted by Morale et al. in Maharashtra, it was reported that literate subjects had better knowledge, positive attitudes, and healthy practices related to dog bite and its management [20]. This study also indicated similar results with respect to knowledge. A similar study done in Shone Town in Southern Ethiopia also reported similar results [21]. A Bangladeshi study was also suggestive of education status being significantly associated with awareness of rabies [22].

The treatment-seeking behavior in this study population was considered good if the victim sought treatment within 24 hours of animal bite exposure. It was found to be good among 68% of the study participants, whereas the rest had poor practices owing to a delay in seeking healthcare. In a study coducted by Sahni et al. in Amritsar it was reported that around 98.3% of the participants were bitten by dogs, 51% of them washed the wound after the bite, and around 70% of the participants reported to the anti-rabies clinic, whereas 21% resorted to traditional healers, but 31% of them received the vaccine on the first day [23]. In a similar study done in Aurangabad by Chopade, around 66% of the study participants reported to a health facility within 24 hours of exposure to an animal bite for treatment [24]. This finding is consistent with the findings of this study. Similar results were reported in a predictive cross-sectional study by Ayub et al. in Patna [25].

As a recommendation, there needs to be an extensive exercise to raise awareness amongst the urban slum residents, as they are most vulnerable to stray animals and stay in close proximity to them. Understanding the course of the disease and identification of the category of the wound can improve the practices related to animal bites. The fact that seeking treatment as soon as possible can be life-saving needs to be reinforced in order to have appropriate health-seeking behavior rather than wasting the golden hour visiting traditional healers.

Limitations

This study, being cross-sectional in nature, has its own limitations with respect to causality. The study was conducted amongst urban slum dwellers; hence, the results cannot be generalized to the general public owing to their living conditions. The chances of recall bias cannot be completely eliminated, as the respondents might not remember the category of the bite, though animal bite exposure can rightly be remembered as it is infrequent.

## Conclusions

It was evident from the study that the educational and socioeconomic status of the respondents had a significant association with awareness regarding rabies. Several factors were identified as influencing practices following an animal bite, like first aid after a bite, place of seeking treatment, pet ownership, and identification of the bite category. Although the majority of the study participants sought treatment within 24 hours of exposure, owing to the fatality of the disease, the proportion was relatively low. Other factors, like actions taken if the pet was rabid, actions taken for the biting dog, and timing of ARV, were also some of the animal factors that were significantly associated with the practices following an animal bite. Therefore, an extensive exercise to raise awareness and promote health-seeking behavior has been planned for residents in the study area as a small step toward the rabies eradication strategy in India.

## Appendices

Appendix A

**Table 3:** Questionnaire OBC: Other backward class; SC: Scheduled caste; ST: Scheduled tribe; PG: Postgraduate; ARV: Anti-rabies vaccine The questionnaire was created by the authors.

Part A: Sociodemographic Details	
Age (in completed years)	
Sex	(a) Male (b) Female (c) Other
Religion	(a) Hindu (b) Muslim (c) Christian (d) Sikh (e) Other __________
Caste	(a) General (b) SC (c) ST (d) OBC
Educational status	(a) Illiterate (b) Below Primary (c) Middle School (d) High School (e) Intermediate (f) Graduate/PG (g) Professional/Honors
Occupation of head of household	(a) Unemployed (b) Street vendor/small informal business (c) Government employee (d) Own business (e) Private employee (f) Other __________
Socioeconomic status (Modified BG Prasad scale)	
Pet ownership	(a) Yes (specify: dog/cat/bird/other) (b) No
Part B: Knowledge and Awareness Regarding Rabies	
1. Have you heard about rabies?	(a) Yes (b) No
2. Source of information (tick all that apply)	(a) Radio/TV (b) Newspaper/books (c) Public meeting (d) Neighbors (e) Parents (f) Doctor/veterinarian (g) Schooling (h) Other __________
3. Which animals can get rabies? (tick all that apply)	(a) Dogs (b) Cats (c) Cows (d) Sheep (e) Goats (f) Pigs (g) Rabbits (h) Humans (i) Jackal (j) Other __________
4. How is rabies transmitted between animals?	(a) Bite (b) Licking wound (c) Scratch (d) Food (e) Licking intact skin (f) Other _________
5. How is rabies transmitted from dogs to humans?	(a) Bite (b) Licking wound (c) Scratch (d) Licking intact skin (e) Do not know
6. Do you know clinical signs of rabies in dogs?	(a) Yes (b) No
7. Signs of rabies in dogs (tick all that apply)	(a) Aggressiveness (b) Profuse salivation (c) Pica (d) Difficulty swallowing (e) Roaming long distances (f) Change in sound (g) Dropping of jaw (h) Other __________
8. Prognosis for rabies in dogs once symptoms appear	(a) Can be treated successfully (b) Always die (c) Do not know
9. Prognosis for rabies in humans once symptoms appear	(a) Can be treated successfully (b) Always die (c) Do not know
10. Most effective rabies control method in dogs	(a) Killing stray dogs (b) Restriction of movement (c) Vaccination (d) Castration
11. Symptoms of rabies in humans (tick all that apply)	(a) Frothing at mouth (b) Hydrophobia (c) Abnormal behavior/madness (d) Do not know
12. Best way to control dog-mediated rabies in humans	(a) Vaccination of dogs (b) Restriction of dogs (c) Public education (d) Killing stray dogs (e) Vaccination of high-risk groups (f) Post-exposure prophylaxis
13. What would you do if your pet became rabid?	(a) Consult veterinarian (b) Kill the animal (c) Vaccinate (d) Isolate (e) Do not know
14. Do you know about the rabies vaccine (ARV)?	(a) Yes (b) No
15. When should ARV be taken?	(a) After exposure (b) Before exposure (c) Both
16. Site of ARV injection	(a) Abdomen (b) Shoulder (c) Thigh (d) Site of bite (e) Do not know
17. Do you know about rabies immunoglobulin (RIG)?	(a) Yes (b) No
18. Is rabies a preventable disease?	(a) Yes (b) No (c) Do not know
19. Is rabies always fatal after symptoms appear?	(a) Yes (b) No (c) Do not know
20. Should all animal bites be treated?	(a) Yes (b) No (c) Do not know
21. Can rabies spread by minor scratches without bleeding?	(a) Yes (b) No (c) Do not know
22. Can rabies spread by saliva contact on broken skin?	(a) Yes (b) No (c) Do not know
23. Is wound washing important after dog bite?	(a) Yes (b) No (c) Do not know
24. Is rabies vaccine available free of cost in government hospitals?	(a) Yes (b) No (c) Do not know
25. Is pre-exposure vaccination recommended for veterinarians and pet handlers?	(a) Yes (b) No (c) Do not know
26. Can rabies be transmitted by licking of intact skin?	(a) Yes (b) No (c) Do not know
27. Is there a law for pet registration in your area?	(a) Yes (b) No (c) Do not know
Part C: Practices After an Animal Bite	
1. What did you do immediately after an animal bite?	(a) Washed wound with soap/water (b) Used antiseptic (c) Applied salt/oil/herbal remedies (d) Did nothing
2. Where did you seek treatment?	(a) Government hospital (b) Private hospital (c) Household treatment/traditional healer (d) Local quack (e) Did nothing
3. What action was taken for the biting dog?	(a) Observed for 10 days (b) Released if vaccinated (c) Killed immediately (d) Do not know
4. Awareness of ARV timing	(a) After bite/exposure (b) Before exposure only (c) Do not know
5. If your pet is rabid, what should you do?	(a) Consult veterinarian (b) Kill the animal (c) Isolate (d) Do not know
6. Can you recognize which bite categories (II/III) are risky?	(a) Yes (b) No (c) Do not know
